# The Modern Playwright and the Medical Practitioner

**Published:** 1907-02-02

**Authors:** 


### THE MODERN PLAYWRIGHT AND THE MEDICAL PRACTITIONER.

### "THE DOCTOR'S DILEMMA" AT THE COURT THEATRE.

Mr. Bernard Shaw's play with this title, as presented at the Court Theatre, is one which all thoughtful members of the medical profession, as well as of the public, would do well to see. The audience treated the first three acts as a huge joke; but, although the play has in it many things that are humorous, underlying the humour is a keen apprehension of much in the modern world that is going on, which, to say the least, needs reconsideration and the exercise of discipline. To the young, the ignorant, in the sense of knowledge of the world, and to the inexperienced, the play may have little serious meaning, for Mr. Shaw has the unique gift amongst modern playwrights of producing excellent dialogue, which read superficially seems smart and amusing; but for the experienced and informed it contains much which should give cause for thought. Those who have a wide knowledge of life and its meaning must be struck by the keenness of Mr. Shaw's perception, and the daring of his presentation of subjects which rest upon a basis of truth. Mr. Shaw's gifts include also keen insight into the actualities of life, and literary qualities of so high an order, that his plays are as readable, as they are interesting when presented on the stage. Underlying some things, which are scarcely true to art, we find evidence of sound principles, and the remorseless exposure of many shams. The power of terse expression, which Mr. Shaw possesses, gives a charm to most of his work. His defects, as exhibited in "The Doctor's Dilemma," seem to us to include evidence that, his phenomenal success has made him do less than justice to the perception of his readers and audience, whilst his eye seems to be fixed too continuously on the main chance. In the play in question, he has certainly shown a disregard for truth in character, especially in his delineation of Sir Colenso Ridgeon in the last act. This last act seems to fulfil no artistic or other purpose. Its inclusion in the play is a mistake, in our view.

Mr. Shaw is at present the rage with undergraduates at the Universities, who regard him as a brilliant cynic, full of life and wit. That, however, is merely a young man's judgment, and it contains something less than justice, for it fails to recognise that Mr. Shaw as a cynic suggests, as the motto of modern life, the simple legend—

"Just as good as the real."

There is evidence in the play under consideration, that, Mr. Shaw knows and believes in the true as opposed to the false. It is for this reason that we recommend every member of the profession who has the opportunity to go to see "The Doctor's Dilemma."

The play brings out what we assume Mr. Shaw considers the weaknesses and follies of medical men, artists, and members of the Press. The playwright exhibits, from his point of view, the modern Court physician, the honoured and mature consultant of a past generation, the physician who is a scientist and the discoverer of a special toxin, and the general practitioner whose fees by the competition of hospitals and free medical relief agencies have been reduced to starvation point. Of course there is necessarily a considerable element of caricature in the presentation, but those who know most of the profession, as it is to-day, will, we think, find matter for reflection as well as for amusement in the words Mr. Shaw has put into the mouths of his various characters. Surgery has strict limitations, and the use of the knife is a matter which requires control and restraint. The rage for operations, which afflicts numbers of people in the present day, can only be compared to the craze for tattooing, of which the savage still possesses almost a monopoly.

Sir Patrick Cullen, by his bluntness and the sound principles which underlie his words of counsel and criticism, does justice to the large body of practitioners who are also gentlemen. Those who have had the privilege of knowing the great leaders of the profession, during the last fifty years, will recall many instances in consultant practice, as they listen to the last words uttered by Sir Patrick Cullen to Mrs. Dubedat, after her husband's death.

As to the presentation of the artist, whose merits as a painter move Mr. Shaw to eloquence, he represents a type and character of man seldom met with, nowadays, amongst artists who have any claim to that title.

The Press representative would have to be regarded as a burlesque, if it were not unfortunately true that some editors are far too careless in their selection of men, whose chief duty it is to interview people, for the purposes of copy.

Taken as a whole, the wider the experience and knowledge of the workings of the world possessed by the student of this play, the greater will be his appreciation of much that it contains. Unless we misunderstand them, Mr. Shaw's chief doctrines aim at maintaining that virtue is the only good, that the essence of virtue is self-control, and that pleasure is evil if sought for its own sake. Certainly those who wish to take a fair view of Mr. Shaw's attitude might draw these conclusions from much that is contained in "The Doctor's Dilemma." The moral of the play seems to be that what people need nowadays is the exhibition of more intelligence and a closer adherence to principle. It is a great responsibility for a playwright to exhibit the courage and temerity which many of Mr. Shaw's plays possess. That they are able to hold the audience in close attention from the rising of the curtain to its fall, and also continuously to interest the student, who reads the plays with attention in his study, may be taken as proof of the excellence of the workmanship and the genius of the author. Whilst there are some things in "The Doctor's Dilemma" which we regret to see presented on the stage, there is much to inspire thought in the intelligent listener. For these reasons we are of opinion that, all members of the profession—and, indeed, all who have any interest in the modern play—should make it their business to see Mr. Shaw's latest work.

